# Brief Communication: Increased Frequency of Micronucleus in the Bone Marrow of Mice Under Simulated Occupational Exposure to Isoflurane

**DOI:** 10.1002/em.70052

**Published:** 2026-05-11

**Authors:** Marina Zanin, Mariana Gobbo Braz, Maria Vitória Destro, Antonio S. Varela Junior, Martielo Ivan Gehrcke, Carine Dahl Corcini

**Affiliations:** ^1^ Institute of Biological Sciences, Federal University of Rio Grande (FURG) Rio Grande Brazil; ^2^ UNIPEX – GENOTOX Laboratory (Division of Anesthesiology) São Paulo State University (UNESP), Botucatu Medical School Botucatu Brazil; ^3^ RAC—Center for Teaching and Research in Animal Reproduction Faculty of Veterinary Medicine, Federal University of Pelotas Pelotas Brazil; ^4^ Faculty of Veterinary Medicine Federal University of Pelotas (UFPel) Pelotas Brazil

**Keywords:** inhalation anesthetic, mice, micronucleus assay, occupational exposure

## Abstract

Isoflurane is a widely used inhalational anesthetic. Occupational exposure to waste anesthetic gases (WAGs) raises concerns about DNA damage. Bone marrow, highly susceptible due to its proliferative activity, has been scarcely investigated in rodents after exposure to WAGs. Therefore, the present study evaluated the impact of this exposure on micronucleus (MN) frequency and cytotoxicity, under conditions that mimic work environments with WAG to which thousands of professionals worldwide are occupationally exposed. Swiss mice were randomized into control, exposed, and recovery groups. Animals were subjected to daily 5 h exposures to 50 ppm isoflurane for 30 days. Bone marrow samples were analyzed using the MN assay. Isoflurane‐exposed mice showed a significant increase in micronucleated polychromatic erythrocytes compared to controls (*p* < 0.0001). The recovery group exhibited a partial reduction after 20 days without exposure, though values remained above the control. Simulated occupational isoflurane exposure induces chromosomal instability in bone marrow cells, even at recommended safety levels.

## 1 Introduction

The introduction of inhalation anesthetics represented a milestone in anesthetic practice, enabling safe and effective induction and maintenance of general anesthesia. Among these agents, isoflurane (C_3_H_2_ClF_5_O) stands out, a widely used halogenated fluorocarbon whose low blood solubility, rapid anesthetic recovery, and favorable hemodynamic profile differentiate it from other compounds in the same class (Freiermuth et al. 2016). In comparison to the other halogenated anesthetics, such as sevoflurane and desflurane, isoflurane is cheaper.

Despite its established clinical safety profile, concerns remain about the toxic effects of occupational exposure among healthcare professionals working in operating rooms and experimental laboratories, who are frequently exposed to waste anesthetic gases—WAGs (Silva et al. 2025). Although different institutions propose exposure limits, the Occupational Safety and Health Administration (OSHA) recognizes that, given the lack of consistent data on the relationship between exposure and effect, the risk levels of newer halogenated agents, such as isoflurane, cannot be fully established (OSHA 2000). Indeed, previous studies have already demonstrated increased DNA damage (micronucleus—MN in lymphocytes and comet assay in mononuclear cells, respectively) of professionals occupationally exposed to high levels of WAGs, both in mixture or only to isoflurane (Braz, Figueiredo, et al. 2024; Silva et al. 2026).

However, the genotoxicity/mutagenicity of isoflurane remains controversial. While negative results were observed in classical assays, such as the Ames test in vitro model (Baden et al. 1977), in *Drosophila melanogaster* (Kundomal and Baden 1985), and in peripheral lymphocytes of surgical patients anesthetized with isoflurane, as evaluated by the comet assay (Braz et al. 2011), other studies have reported increased frequency of micronucleated kidney cells in rats exposed to isoflurane (Robbiano et al. 1998), damage to DNA by the comet assay in lymphocytes and tissues (such as bone marrow, spleen and liver) of rats (Kim et al. 2006), and increased DNA damage (comet assay) in lymphocytes of patients undergoing surgical procedures (Karabıyık et al. 2001). In addition, isoflurane impairs sperm parameters in mice under both acute (Zanin et al. 2024b) and occupational exposure (Zanin et al. 2024a).

These contradictions reported with isoflurane may be related to variations in the duration and intensity of exposure, the experimental model used, and the sensitivity of the biomarkers applied. However, no study has yet investigated the impact of occupational exposure to WAG isoflurane on bone marrow. This is a highly relevant approach, considering the high susceptibility of bone marrow cells to genotoxic damage and the potential systemic, silent, and lasting effects that such changes can have on all cell lines. Furthermore, it should be considered that genetically altered cells can be eliminated from the peripheral circulation, reducing the detection of genotoxic events in the blood. In the marrow, on the contrary, damage can be identified before this selection process, providing more reliable information for assessing the toxicity of an agent (Vikram et al. 2007).

In this context, animal models play a central role in elucidating toxicity mechanisms. Among the available tools, the MN test is widely used for its sensitivity, reproducibility, and recognition as a standard method (OECD 2016, n. 474) in the assessment of chromosomal damage and cytotoxicity. Investigations in experimental models allow for a better understanding of the risks arising from occupational exposure, providing support for further regulatory and health protection measures for exposed professionals.

Therefore, the present study aimed to evaluate the genotoxic effects of simulated occupational exposure to isoflurane in Swiss mice, using the MN test in bone marrow. The importance of this study is to fill the existing gap on the impacts of this exposure on MN frequency and cytotoxicity, under conditions that mimic work environments with WAG to which thousands of professionals worldwide are occupationally exposed. Additionally, we aimed to assess the bone marrow's capacity for recovery/repair following the interruption of WAG exposure, based on the hypothesis that occupational exposure to WAG isoflurane is detrimental to bone marrow health, whereas its suspension may restore the viability and genetic integrity of these cells. For this purpose, we analyzed the effects after 30 consecutive days of WAG isoflurane exposure, as well as the delayed effects, evaluated 20 days after the final exposure.

## 2 Materials and Methods

### 2.1 Animal Management

All procedures in this study were approved (#36567‐2019) and adhered to the guidelines of the Ethics and Animal Experimentation Committee of the Federal University of Pelotas (UFPel) and followed the regulations of the National Council for the Control of Animal Experimentation (CONCEA).

The experiment took place at the UFPel (Pelotas, Rio Grande do Sul, Brazil). Male Swiss albino mice, aged 6 weeks, were obtained from the institution's vivarium. Inclusion and exclusion criteria were established beforehand to guarantee the selection of animals without anatomical abnormalities. Before the start of the study, the mice were allowed a 1‐week acclimatization period. They were kept in the rodent facility in three polypropylene cages (414 × 344 × 174 mm) containing wood shavings and fitted with stainless steel wire lids, with 10 animals per cage. Environmental conditions were controlled, maintaining a 12 h light/dark cycle, a temperature of 22°C ± 1°C, and a relative humidity of 55% ± 5%. Food and water were provided ad libitum, using commercial pelleted chow formulated for rodents (Presence Labina—rats and mice, Brazil).

### 2.2 Experimental Design

**FIGURE 1:**
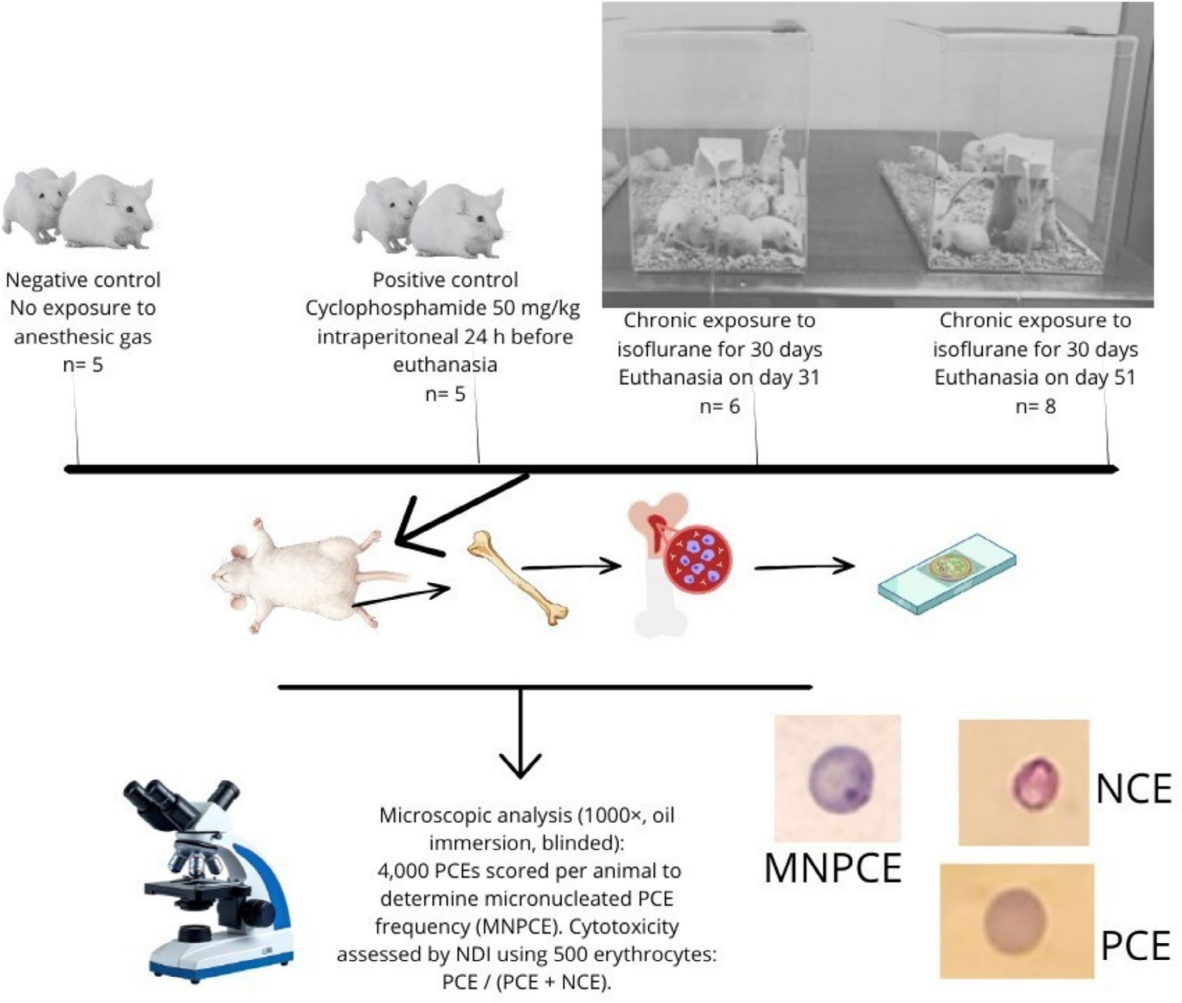
Experimental design of the current study.

Twenty‐four mice were distributed into four experimental groups (Figure 1) by simple randomization. To minimize potential confounding effects, each group's cages were identified using different colors. The negative control group (NCG, *n* = 5) consisted of animals that were not exposed to WAG isoflurane but only to ambient air enriched with pure oxygen for 30 consecutive days and was euthanized on Day 31, and the positive control group (PCG, *n* = 5) was composed of animals that were administered cyclophosphamide at a dose of 50 mg/kg (ip) on the last day of exposure, and euthanasia occurred 24 h after the treatment, on Day 31. Two exposed groups were evaluated, as follows: the first group (EG1, *n* = 6) underwent exposure to WAG isoflurane for 30 consecutive days and was euthanized on Day 31 (respecting 24 h following OECD guidelines #474, 2016) while the second group (EG2, *n* = 8), referred to as the late group, was subjected to the same exposure but remained unexposed for an additional 20 days before euthanasia, to evaluate possible delayed effects (Zanin et al. 2024a). Cyclophosphamide was used as a positive control due to its well‐established clastogenic activity in the in vivo MN assay (Nai et al. 2015).

### 2.3 Exposure Protocol

Each day, the animals were placed for 5 h in three acrylic chambers (NCG/PCG, EG1, and EG2) with a volume of 21.6 L, fitted with inlet and outlet openings for gas circulation, for over a month. During exposure, the chambers contained wood shavings, and a pure oxygen flow of 1 L/min per box was continuously provided. One chamber functioned as the control, housing animals exposed only to ambient air increased by the flow of pure oxygen. In the remaining chambers, designated as the exposure groups, a drip system was installed. This apparatus released one microdrop (5 μL) of isoflurane onto a gauze pad every 10 min, ensuring vaporization and maintaining a WAG concentration of approximately 50 ppm (American Conference of Governmental Industrial Hygienist [ACGIH] 2022), following a previous protocol (Zanin et al. 2024a). At the end of each daily session, the chambers were opened, oxygen flow was discontinued, and the mice were returned to their housing boxes in the vivarium until the next day's exposure. The regimen was maintained for 30 consecutive days. At the end of the study, euthanasia was carried out by cervical dislocation by an experienced professional 24 h after the end of the exposure/treatment (NCG, PCG, and EG1), and femurs were collected, except for the “late group” (EG2), to observe possible repair (unexposed for 20 days before euthanasia).

### 2.4 MN Test in Bone Marrow

Slides were previously coded, and the MN test was performed following the protocol of MacGregor et al. (1987), with some modifications (Destro et al. 2025). Bone marrow was collected from femurs and smears were prepared, fixed with methanol (Merck, Germany), and stained with Giemsa (Merck). After preparation of all coded slides, microscopic analysis (1000×, oil immersion) was conducted in a blinded manner by an experienced evaluator to score 4000 polychromatic erythrocytes (PCEs) per animal, determining the frequency of micronucleated PCEs (MNPCEs). Cytotoxicity was evaluated by calculating the nuclear division index (NDI) from 500 erythrocytes using the ratio PCE/(PCE + NCE).

### 2.5 Statistical Analysis

The sample size was based on OECD guidelines for MN in mammals (n. 474, 2016). Data distribution was assessed using the Ryan–Joiner normality test. Results from MN were analyzed using generalized linear models applying a Poisson distribution. When significant differences were detected, pairwise post hoc comparisons with Bonferroni correction were performed. For the NDI, comparisons among groups were carried out using one‐way analysis of variance (ANOVA). Differences between groups were considered significant when *p* < 0.05.

## 3 Results

Analysis of the MN test in the bone marrow of Swiss mice (Table 1) revealed a significant increase in MN frequency in both groups exposed to isoflurane (EG1 and EG2), showing a higher number of MNPCEs compared to the NCG (*p* < 0.0001). Animals euthanized on the 31st day of exposure (EG1) had the highest frequency of MNPCEs (0.49%). On the other hand, animals euthanized on the 51st day (EG2), after a 20‐day recovery period without exposure, exhibited a lower frequency of MNPCEs (0.28%) than the other exposed group (EG1), but the frequency was higher than that of the NCG. The NDI remained statistically similar between groups (*p* > 0.05). As expected, the PCG validated the sensitivity of the assay, presenting the highest frequency of MN (0.89%).

**TABLE 1:** Frequency of micronucleated polychromatic erythrocytes (MNPCEs) and nuclear division index (NDI) in the bone marrow of mice exposed or not to waste anesthetic isoflurane.

Groups	No. of animals	No. of analyzed cells	MNPCEs			NDI = PCE/(PCE + NCE), mean ± SD	*p* vs. NCG
			No.	%	Mean ± SD/4000		
NCG	5	20,000	12	0.06^a^	2.40 ± 2.30	0.40 ± 0.08	—
EG1	6	24,000	118	0.49^c^	19.67 ± 6.25	0.50 ± 0.08	< 0.001
EG2	8	32,000	90	0.28^b^	11.25 ± 3.77	0.51 ± 0.06	< 0.001
PCG	5	20,000	177	0.89^d^	35.40 ± 3.58	0.47 ± 0.12	< 0.001

*Note:* Groups represented by different letters differ significantly for MNPCE frequency (Poisson distribution, *p* < 0.0001). Post hoc multiple comparisons with Bonferroni correction were performed, and adjusted *p* values versus the negative control group are presented.

Abbreviations: EG1, animals exposed to traces of isoflurane for 30 days and euthanized on Day 31; EG2, animals exposed to traces of isoflurane for 30 days and remained unexposed for 20 days before euthanasia; NCE, normochromatic erythrocytes; NCG, negative control group; PCE, polychromatic erythrocytes; PCG, positive control group.

## 4 Discussion

This study is the first to demonstrate that the adverse effect of occupational exposure to WAG isoflurane on bone marrow is a significant increase in the frequency of MNPCEs in mice, evidencing genotoxic damage in this tissue. Experimental exposure was performed at a concentration of 50 ppm, a value established by the ACGIH as a safe occupational exposure limit (ACGIH 2022), despite different international recommendations existing with different thresholds.

Considering no studies are available in this theme, making a parallel with a study that evaluated an acute exposure to isoflurane (30 or 60 min) reported oxidative damage to DNA (comet assay), lipids, and proteins in different tissues of rats, including bone marrow (Kim et al. 2006), similarly to our findings. Drawing a parallel with a clinical study, Karabıyık et al. (2001) observed a significant increase in DNA damage in lymphocytes from patients anesthetized with isoflurane for 60 or 120 min of anesthesia and on the first day; removal of the DNA damage was observed after the third day of anesthesia, and the repair was completed within 5 days. Interestingly, veterinarians exposed to high levels of WAG isoflurane showed increased frequency in MN evaluated both in oral exfoliated cells and lymphocytes (Figueiredo et al. 2022; Braz, Figueiredo, et al. 2024). Notably, dosimeter data indicated high levels of WAG exposure by veterinarians, and a possible biomarker of WAG isoflurane exposure, an epoxyoctadecenoic acid (EpOME), was identified in plasma by a metabolomics approach (Silva et al. 2026). In addition, recent evidence shows that the same environmental concentration of isoflurane applied in the present study impaired reproduction and sperm genetic damage in mice (Zanin et al. 2024a), supporting that different tissues undergoing cell division may be targets of this exposure.

Contrarily, a study suggested that, in contrast to sevoflurane exposure, acute exposure to isoflurane did not induce DNA damage (comet assay) in the blood of rats (Rocha et al. 2015). Some clinical studies conducted in surgical patients exposed to isoflurane did not observe increased DNA damage (comet assay) or apoptosis in patients' lymphocytes (Braz et al. 2011; Braz, Silva, et al. 2024). Negative findings of the comet assay and absence of apoptosis in lymphocytes were reported by Souza et al. (2021) in healthcare professionals exposed to a mixture of WAGs, including isoflurane. In addition, occupational exposure to WAGs below recommended levels was not associated with changes in MN frequency in buccal cells of anesthesiologists (Braz et al. 2020).

In the present study, the choice of bone marrow as the target for evaluation is particularly relevant, considering its high proliferative rate and consequent susceptibility to genotoxic events. The increase in MN observed occurred without any change in the NDI, indicating that proliferation was not inhibited and that the damage detected was not due to cytotoxicity. This finding suggests that cells carrying chromosomal alterations remain viable and may have a systemic impact on exposed animals.

Regarding the potential for recovery after cessation of exposure, our results indicate that bone marrow exhibits a partial repair capacity during the evaluated period. Although animals subjected to delayed euthanasia showed a significant reduction in the number of damaged cells compared with the immediate analysis, the values remained higher than those observed in the NCG. These findings contrast with the observations of Karabıyık et al. (2001), who reported normalization of DNA damage (comet assay) in lymphocytes from patients a few days after anesthesia with isoflurane. In bone marrow, MN detected in PCEs reflect chromosomal damage occurring approximately 24–48 h before sample collection (OECD 2016, n. 474). Therefore, when the genotoxic insult ceases and erythropoiesis returns to normal, MN frequencies are expected to gradually decline as damaged cells are replaced through normal erythroid turnover. Persistent elevation of MN after cessation of exposure is uncommon and rarely reported. One example has been described following ionizing radiation exposure in p53‐deficient mice (Chang et al. 2000), which may suggest that mechanisms associated with impaired genomic stability may contribute to sustained MN formation. Considering the high proliferative rate and turnover dynamics of bone marrow cells, a plausible explanation is that WAG isoflurane exposure may affect genomic stability mechanisms in hematopoietic precursor cells. In addition to its reported effects on oxidative stress (Kim et al. 2006; Silva et al. 2026), isoflurane may also influence gene expression patterns related to DNA repair and apoptosis pathways (Braz et al. 2011), potentially disrupting processes involved in DNA replication fidelity or chromosome segregation. Such alterations could lead to increased spontaneous MN formation even after cessation of exposure.

Overall, our results indicate that (sub)chronic occupational exposure to WAG isoflurane, even at concentrations considered safe by regulatory agencies, such as ACGIH (2022), but not the National Institute of Occupational Safety and Health (NIOSH 1977), can induce genomic instability in dividing cells, with persistent effects. The detection of MN reinforces this scenario, as these DNA fragments or chromosomes not incorporated into the main nucleus are widely recognized as a biomarker of mutagenesis. Thus, this study reinforces the need for a critical review of current occupational exposure recommendations, including those proposed by OSHA, CDC (Garcia et al. 2022), and Yale University (2023), which follow the ACGIH guideline of 50 ppm as time‐weighted average as a safety limit.

Some limitations should be considered. The animal model, although it allows rigorous experimental control, does not fully reproduce the complexity of human exposure, which can be intermittent and eventually of lower intensity. In addition, the analysis focused on specific biomarkers; the inclusion of additional parameters, such as systemic oxidative stress and epigenetic changes, could broaden the understanding of the risks involved.

In summary, this is the first study to demonstrate the genotoxic effect (increased MN frequency) of simulated occupational exposure to WAG isoflurane in bone marrow at concentrations equivalent to the ACGIH recommended limit. The results indicate that this anesthetic agent, widely used in hospital and laboratory settings, poses a potential risk of inducing genomic instability, with possible long‐term repercussions. These findings reinforce the urgency of improving biosafety measures, reevaluating exposure parameters considered safe, and developing longitudinal studies in humans that can elucidate the real impacts of chronic exposure to WAGs.

## Author Contributions

**Marina Zanin:** conceptualization, experiment execution, formal analysis, investigation, methodology, roles writing – original draft, writing – review and editing. **Mariana Gobbo Braz:** conceptualization, methodology, supervision, validation, writing – review and editing. **Maria Vitória Destro:** methodology, formal analysis, writing – review and editing. **Antonio S. Varela Junior:** conceptualization, methodology, project administration, supervision, validation – visualization, writing – review. **Martielo Ivan Gehrcke:** conceptualization, methodology, supervision, validation – visualization. **Carine Dahl Corcini:** conceptualization, resources – funding acquisition, methodology, project administration, supervision, validation, writing – review and editing.

## Funding

This work was supported by Coordenação de Aperfeiçoamento de Pessoal de Nível Superior and Conselho Nacional de Desenvolvimento Científico e Tecnológico (#401750/2023‐0 and #305231/2021‐9).

## Conflicts of Interest

The authors declare no conflicts of interest.

## Data Availability

The data that support the findings of this study are available from the corresponding author upon reasonable request.
